# Low Vitamin D Levels Predict Mortality in Ankylosing Spondylitis Patients: A Nationwide Population-Based Cohort Study

**DOI:** 10.3390/nu12051400

**Published:** 2020-05-13

**Authors:** Niv Ben-Shabat, Abdulla Watad, Aviv Shabat, Nicola Luigi Bragazzi, Doron Comaneshter, Arnon D. Cohen, Howard Amital

**Affiliations:** 1 Sackler Faculty of Medicine, Tel-Aviv University, Tel Aviv-Yafo 6997801, Israel; nivben7@gmail.com (N.B.-S.); Howard.Amital@sheba.health.gov.il (H.A.); 2 Department of Medicine ‘B’, The Zabludowicz Center for Autoimmune Diseases, Sheba Medical Center, Ramat Gan 5265601, Israel; 3 NIHR Leeds Musculoskeletal Biomedical Research Unit, Section of Musculoskeletal Disease, Leeds Institute of Molecular Medicine, Chapel Allerton Hospital, University of Leeds, Leeds LS7 4SA, UK; 4 Hadassah Faculty of Medicine, The Hebrew University, Jerusalem 9112001, Israel; aviv.shabat@gmail.com; 5 Laboratory for Industrial and Applied Mathematics (LIAM), Department of Mathematics and Statistics, York University, Toronto, ON M3J 1P3, Canada; robertobragazzi@gmail.com; 6 Chief Physician’s Office, Clalit Health Services Tel Aviv, Tel-Aviv 6209813, Israel; doronko1@clalit.org.il (D.C.); arcohen@clalit.org.il (A.D.C.); 7 Siaal Research Center for Family Medicine and Primary Care, Faculty of Health Sciences, Ben Gurion University of the Negev, Beer Sheva 8410501, Israel

**Keywords:** vitamin D, ankylosing spondylitis, mortality, 25-hydroxyvitamin D, Autoimmunity

## Abstract

In this study, we aimed to examine the effect of vitamin D deficiency on all-cause mortality in ankylosing spondylitis (AS) patients and in the general population. This is a retrospective-cohort study based on the electronic database of the largest health-maintenance organization in Israel. AS patients who were first diagnosed between 2002–2007 were included. Controls were matched by age, gender and enrollment-time. Follow-up continued until death or end of study follow-up on 1 July 2019. Laboratory measures of serum 25-hydroxyvitamin-D levels during the entire follow-up period were obtained. A total of 919 AS patients and 4519 controls with a mean time of follow-up of 14.3 years were included. The mean age at the time of enrollment was 52 years, and 22% of them were females. AS was associated with a higher proportion of vitamin D deficiency (odds ratio 1.27 [95% confidence-interval (CI) 1.03–1.58]). In AS patients, insufficient levels of vitamin D (<30 ng/mL) were significantly associated with increased incidence of all-cause mortality (hazard ratio (HR) 1.59 [95% CI 1.02–2.50]). This association was more prominent with the decrease in vitamin D levels (< 20 ng/mL, HR 1.63 [95% CI 1.03–2.60]; <10 ng/mL, HR 1.79 [95% CI 1.01–3.20]) and among male patients (<30 ng/mL, HR 2.11 [95% CI 1.20–3.72]; <20 ng/mL, HR 2.12 [95% CI 1.19–3.80]; <10 ng/mL, HR 2.23 [95% CI 1.12–4.43]). However, inadequate levels of vitamin D among controls were not associated with an increased all-cause mortality. Our study has shown that vitamin D deficiency is more common in AS patients than controls and is linked to an increased risk for all-cause mortality. These results emphasize the need for randomized-controlled trials to evaluate the benefits of vitamin D supplementation as a secondary prevention of mortality in patients with chronic inflammatory rheumatic disease.

## 1. Introduction

Vitamin D is classically known to play a pivotal role in regulating calcium metabolism, but in recent years there is a growing body of evidence for its function as an immunomodulator of both the innate and adaptive immune system [1,2,3,4,5,6]. Association between circulating levels of vitamin D, disease occurrence and severity was found in several immune conditions, such as multiple sclerosis (MS) [7], type I diabetes mellitus [8], inflammatory bowel disease (IBD) [9], autoimmune thyroid diseases [10], systemic lupus erythematous (SLE) [11,12], systemic sclerosis [13] and rheumatoid arthritis (RA) [14].

The prevalence of vitamin D deficiency is estimated to be between 20–100%, depending on ethnicity, geographical location and season [15,16]. However, the association between vitamin D status and mortality is still awaiting elucidation. Several studies reported an inverse association between serum levels of 25-(OH)-hydroxyvitamin D [25(OH)D] and mortality, in particular cardiovascular- and cancer-associated mortality [17,18]. Yet, previous studies have shown no benefit from vitamin D supplementation in terms of survival rate [19], or in reducing the incidence of cancer and cardiovascular disease [20].

Studies addressing chronic inflammatory rheumatic diseases (CIRD) showed an inverse association between serum 25(OH)D levels, the incidence, and disease severity of rheumatoid arthritis (RA), Bechet disease, systemic sclerosis and psoriatic arthritis [21,22,23,24].

In ankylosing spondylitis (AS) patients, previous studies reported lower serum levels of 25(OH)D compared to controls [14,25,26,27,28,29,30,31]. With respect to the effect of vitamin D status on disease severity, reported results are controversial, with some studies showing that vitamin D deficiency increases the disease severity in AS patients [27,28,32], while other investigations report no association [14,31,33]. Most of these studies were cross-sectional studies, had a small sample size and none of them have investigated the impact of vitamin D levels on mortality in AS patients to date. Therefore, we have investigated the association between AS and vitamin D deficiency and the impact of vitamin D serum levels on mortality rates among patients with AS, in a large-scale, population-based study.

## 2. Materials and Methods 

### 2.1. Data Source

Data were obtained from the Clalit Healthcare Services (CHS) comprehensive, electronic database. CHS is the largest health maintenance organization in Israel and serves approximately 4.4 million insured members (over 50% of the Israeli population) from heterogeneous ethnic groups and receives continuous real-time input data from pharmaceutical, medical and administrative operating systems. The database was designed for the purposes of administrative and clinical management and is available for use in epidemiological studies. Within the database, diagnoses are going through a constant process of validation by logistic checks (such as matching the diagnoses from different sources). These diagnoses were found to be highly reliable, as shown in previously published studies [34,35,36,37,38].

### 2.2. Sample and Design

This study was designed as a retrospective-cohort study. Utilizing the CHS’s computerized database, we extracted a cohort of AS-patients, first diagnosed between 1 January 2002 to 31 December 2007, and compared them with age- and gender-matched controls. With respect to AS patients, the follow-up began on the date of first AS diagnosis and was matched for controls. The follow-up continued until death of the subject or otherwise was ended on 1 July 2019.

### 2.3. Measures

A patient was defined as having AS if he had at least one documented diagnosis of AS in his medical records as an outpatient, either by a primary care physician or a specialist, or if he was diagnosed with AS in hospital discharge papers. Patients under the age of 18 at the time of diagnosis were excluded. Controls were randomly assigned from the CHS database, with the exclusion of AS patients. Approximately five age- and gender-matched controls for each AS patient were included in the study.

Data available from the CHS database included an array of variables, such as age, gender, socioeconomic status (SES), body-mass index (BMI), chronic diseases and laboratory tests. 

SES was defined according to a poverty index based on the patient’s residence area. The poverty index was defined during the 2008 National Census, and considered average household income, education, crowding, and car ownership. We divided the population into three categories based on quartiles (low-25 percentile; medium 25–75 percentile; high 75–100 percentile). 

BMI was calculated using height and weight measures at the time of enrollment to the study (if available). BMI was classified into four categories: underweight, <18.5 kg/m^2^; normal, 18.5–24.9 kg/m^2^; overweight, 25–30 kg/m^2^; obese >30 kg/m^2^. The normal category was used as a reference category.

Patient was defined as having cardiovascular disease (CVD) if he had at least one documented diagnosis of ischemic heart disease, cerebrovascular disease or peripheral vascular disease in his medical records. In the same manner, malignancy was defined based on a diagnosis of any solid or hematological malignancies.

Vitamin D status was assessed using serum measurements of 25(OH)D. For each subject, mean levels of all laboratory measurements of 25(OH)D during the follow-up were calculated and classified into four categories: severe deficiency, ≤ 10 ng/mL; deficiency, ≤ 20 ng/mL; insufficiency, 21–29 ng/mL; and adequate ≥30 ng/mL [16]. The adequate category was used as a reference category.

### 2.4. Statistical Analysis

Differences in baseline characteristics between different groups of independent variables were compared using *t*-test or Mann–Whitney U test for continuous variables, and χ^2^ test for categorical variables. 

The association between AS and vitamin D deficiency was evaluated by a standard unconditional logistic regression model, adjusted for age and gender.

Survival analysis using multivariate Cox proportional hazards method was performed to detect association between vitamin D deficiency with an increased risk of all-cause mortality, separately for AS patients and controls. The first model was adjusted for age and gender while the second was adjusted for CVD and malignancy as well. 

Statistical analysis was performed using the commercial software “Statistical Package for the Social Sciences” (SPSS for Windows, V.23.0, IBM SPSS Statistics, Armonk, NY, USA).

### 2.5. Ethics

This study was approved by the CHS Ethics Committee in Tel Aviv, Israel. Approval number was 0212-17-COM. No informed consent was needed (existing database).

## 3. Results

### 3.1. Cohort Characteristics

A total sample of 5438 subjects (4519 controls and 919 AS patients) was included. No significant difference was found between AS patients and controls in terms of age at the time of study enrollment (52.3 ± 15.9 years in AS vs. 52.1 ± 15.9 years in controls). The median follow-up time among the entire cohort was 14.3 ± 3.9 years and was significantly higher in controls compared to AS patients (15.4 years vs. 15.6, *p* < 0.05). The female proportion was similar in both study groups (22.7% in AS vs. 22.9% in controls, *p* = 0.96). Furthermore, AS patients and controls did not differ in terms of SES and BMI Rates of CVD (36% vs. 30.9%, *p* < 0.001) and malignancy (24.4% vs. 16.9%, *p* < 0.001) were significantly higher in AS patients (Table 1). 

### 3.2. Vitamin D Status

The serum levels of 25(OH)D (ng/mL) throughout the follow-up were significantly lower among AS patients compared to controls (mean ±SD) (20.94 ± 9 ng/mL in AS vs. 21.65 ± 8.8 ng/mL in controls; *p* < 0.05). As the levels of 25(OH)D do not have a normal distribution curve, median (IQR) levels were compared as well, showing similar results (20.6 (14–26) ng/mL in AS vs. 21.3 (15–27) ng/mL in controls; *p* < 0.05). AS was significantly associated with a higher proportion of vitamin D deficiency and severe deficiency (< 20 ng/mL, odds-ratio, OR 1.27 [95% CI 1.03–1.58]; <10 ng/mL, OR 1.42 [95% CI 1.07–1.89]) (Table 2).

### 3.3. Vitamin D Deficiency and All-Cause Mortality

In AS patients, insufficient levels of vitamin D (< 30 ng/mL) were significantly associated with an increased incidence of all-cause mortality (hazard ratio, HR 1.59 [95% CI 1.02–2.50]). This association was more prominent with the decrease in circulating levels of vitamin D (< 20 ng/mL, HR 1.63 [95% CI 1.03–2.60]; <10 ng/mL, HR 1.79 [95% CI 1.01–3.20]) and for male patients ( < 30 ng/mL, HR 2.11 [95% CI 1.20–3.72]; <20 ng/mL, HR 2.12 [95% CI 1.19–3.80]; <10 ng/mL, HR 2.23 [95% CI 1.12–4.43]). The trend did not change when accounting for malignancy and CVD. In controls, inadequate levels of vitamin D, regardless of severity, were not associated with increased all-cause mortality (< 30 ng/mL, HR 0.94 [95% CI 0.79–1.11]; <20 ng/mL, HR 0.96[95% CI 0.80–1.14]; <10 ng/mL, HR 1.06 [95% CI 0.82–1.35]) (Table 3).

## 4. Discussion

In this large, nationwide, retrospective cohort study, we found a significant association between AS and low serum levels of vitamin D. In addition, low levels of serum 25(OH)D (< 30 ng/mL) were significantly associated with increased all-cause mortality in AS patients. The strength of this association was more prominent with the severity of vitamin D deficiency and in male patients. In addition, the association remained significant when adjusting for the presence of CVD and malignancy, the two most common causes of death in the western populations. In controls, no association between vitamin D deficiency and increased all-cause mortality was found.

To the best of our knowledge, this is the first study to examine the impact of circulating vitamin D levels on the mortality rate in AS patients, yet several studies have looked at the link between AS, vitamin D status and disease activity [14,24,25,26,27,28,29,30,31,32,33]. In line with our findings, most of these studies reported lower serum levels of 25(OH)D in AS patients than in controls [24,25,26,30,31,32], and an inverse association with disease activity [24,25,27,28,32]. 

Several studies, including systematic reviews, have previously investigated the association between circulating levels of 25(OH)D and all-cause mortality in the general population [17,39,40,41,42,43,44]. In contrast to our findings, most of these studies reported an increased mortality rate in subjects with vitamin D deficiency. However, all these studies excluded participants under the age of 50 years and had a significantly older population than the population in our study, with a median age of 63 years (IQR 59–71) [17]. Therefore, these findings are not adequately comparable. 

In our study, vitamin D deficiency was a predictor of mortality in AS patients, especially among male patients. This finding is supported by the results of a recently published Cochrane systematic review on vitamin D supplementation for the prevention of mortality, which suggests a differential effect of vitamin D across genders [45].

Plausible mechanisms that can explain the increased mortality in AS patients with vitamin D deficiency may be related to the role of vitamin D as an immunomodulator. Vitamin D suppresses adaptive immunity by down-regulating the antigen-presenting activity of macrophages to lymphocytes and shifting the balance of helper T cells from T-helper 1 to T-helper 2, T_reg_ and natural killer T cells [3,21,46,47,48]. Moreover, it has been found that vitamin D inhibits the synthesis of several cytokines, including tumor necrosis factor alpha (TNF-α) which has a key role in joint inflammation and in extra-articular involvement in rheumatic conditions [21,48]. In addition, vitamin D mediates numerous biological functions through the nuclear vitamin D receptor (VDR) known to be highly expressed in immune cells [49]. A recent randomized controlled trial (RCT) conducted in RA patients demonstrated vitamin D anti-inflammatory effect, by showing a significant decrease in inflammatory markers in patients treated with vitamin D supplementation [50]. 

Another aspect that may explain the increased mortality in AS might be related to the enhanced atherosclerosis in such patients, attributed to the chronic inflammatory state and the higher rate of traditional cardiovascular risk factors [51,52,53]. Perhaps vitamin D deficiency, which creates pro-inflammatory state, acts in an additive manner in these patients, as opposed to controls.

The main finding of our study is that vitamin D deficiency is linked to increased all-cause mortality in patients with AS, which may have implications on the potential benefit of vitamin D supplementation as a secondary prevention measure in these patients. Several RCTs were conducted to assess the survival benefit of vitamin D supplementation, reporting inconclusive results [19,45]. Most of these studies only included patients over the age of 50 years, investigated primary prevention strategy and reported results related to the general population and patients with cardiovascular diseases or cancer. Our findings suggest that perhaps a targeted approach, aiming at specific patients such as those with AS who have proven vitamin D deficiency may confer positive outcomes.

Our study has several strengths, including the use of a large and validated database, a heterogeneous population-based cohort, a large sample-size and a long-term follow-up. Nonetheless there are several important limitations that should be acknowledged. First, data regarding AS disease activity such as joint counts, the extent of axial involvement and extra-articular manifestations were lacking. Some variables such as BMI were available only for a proportion of the entire sample. Furthermore, we could not account for the effects of vitamin D dietary intake and seasonality. Moreover, our study design cannot to infer any causality. Finally, we could not adjust for many potential confounders, such as genetic background, and co-morbidities, like chronic renal failure and sarcoidosis.

## 5. Conclusions

Our study found that vitamin D deficiency is more common in AS patients than in controls and is associated with an increased risk for all-cause mortality in AS patients but not in controls. These results underline the need for RCTs examining the impact of vitamin D supplementation on the mortality rate of patients with chronic inflammatory rheumatic disease.

## Figures and Tables

**Table 1:** Baseline characteristics of the study population.

Baseline Characteristics	AS Patients (*n* = 919)	Controls (*n* = 4519)	*p*-Value
Age at enrollment, mean ± SD; median	52.31 ± 15.9; 51.1	52.06 ± 15.9; 50.9	0.67
Time of follow-up (years), median (IQR)	15.4 (14.5–16.5)	15.6 (14.6–16.6)	< 0.05
Gender (female; *n* %)	210 (22.7%)	1028 (22.9%)	0.96
Socioeconomic status ^a^, *n* (%)			0.40
Low	110 (12.7%)	615 (15.6%)	-
Medium	619 (71.7%)	2980 (70.3%)	-
High	134 (15.5%)	644 (15.2%)	-
Body Mass Index ^b^, *n* (%)			0.50
18.5–24.9 kg/m^2^	14 (19.7%)	54 (23.0%)	-
25–29.9 kg/m^2^	19 (26.8%)	75 (31.9%)	-
≥30 kg/m^2^	38 (53.5%)	104 (44.3%)	-
Cardiovascular disease, *n* (%)	331 (36.0%)	1397 (30.9%)	< 0.001
Malignancy, *n* (%)	224 (24.4%)	763 (16.9%)	< 0.001

^a^ Available for 93.7% of data. ^b^ Available for 15.9% of subjects. Abbreviations: AS, ankylosing spondylitis; SD, standard deviation; IQR, interquartile range.

**Table 2:** 25-hydroxyvitamin-D (25[OH]D) levels and rates of vitamin D deficiency in ankylosing spondylitis (AS) patients compared to controls.

25(OH)D Levels (ng/mL)	AS Patients	Controls	OR ^a^ (95% CI)	*p*-Value
Median (IQR)	20.6 (14–26)	21.3 (15–27)	-	<0.05
<10, *n* (% ^b^)	103 (11.2%)	421 (9.4%)	1.42 (1.07 to 1.89)	<0.05
<20, *n* (%)	479 (52.1%)	2187 (48.4%)	1.27 (1.03 to 1.58)	<0.05
<30, *n* (%)	792 (86.2%)	3780 (83.6%)	1.22 (0.99 to 1.49)	0.06

^a^ Relative to proportion of patients with levels ≥30 ng/mL ^b^ data available for 15.9% of subjects. Abbreviations: AS, ankylosing spondylitis; OR, odds ratio; CI, confidence interval; SD, standard deviation; IQR, interquartile range.

**Table 3:** Age- and gender-adjusted hazard ratios (HRs) for all-cause mortality for different levels of serum 25(OH)D.

		AS Patients		Controls	
Population	Serum levels of 25(OH)D (ng/mL) ^a^	Age-and-Gender Adjusted HR (95% CI)	Multivariate ^b^ HR (95% CI)	Age-and-Gender Adjusted HR (95% CI)	Multivariate ^b^ HR (95% CI)
Overall	< 10	1.79 (1.01–3.20)	1.75 (0.97–3.15)	1.06 (0.82–1.35)	1.11 (0.87–1.43)
	< 20	1.63 (1.03–2.60)	1.63 (1.02–2.59)	0.96 (0.80–1.14)	0.99 (0.83–1.18)
	< 30	1.59 (1.02–2.50)	1.61 (1.03–2.53)	0.94 (0.79–1.11)	0.96 (0.81–1.14)
Males	< 10	2.23 (1.12–4.43)	1.99 (0.99–4.01)	1.02 (0.76–1.36)	1.03 (0.77–1.38)
	< 20	2.12 (1.19–3.80)	2.03 (1.13–3.64)	0.97 (0.79–1.19)	0.98 (0.80–1.21)
	< 30	2.11 (1.20–3.72)	2.08 (1.18–3.69)	0.93 (0.77–1.30)	0.94 (0.78–1.15)
Females	< 10	1.11 (0.32–3.84)	1.05 (0.28–4.00)	1.12 (0.68–1.84)	1.33 (0.80–2.21)
	< 20	0.78 (0.35–1.71)	0.80 (0.36–1.78)	0.89 (0.62–1.27)	0.99 (0.70–1.42)
	< 30	0.71 (0.33–1.50)	0.73 (0.34–1.56)	0.92 (0.66–1.29)	1.01 (0.72–1.41)

^a^ Relative to levels ≥30 ng/mL ^b^ Adjusted for age, gender, cardiovascular disease and malignancy. Abbreviations: AS, ankylosing spondylitis; HR, hazard ratio; CI, confidence interval.
